# Clinical relevance of a newly developed endometrial receptivity test for patients with recurrent implantation failure in Japan

**DOI:** 10.1002/rmb2.12444

**Published:** 2022-02-07

**Authors:** Yasuhiro Ohara, Hidehiko Matsubayashi, Yosuke Suzuki, Yukiko Takaya, Kohei Yamaguchi, Masakazu Doshida, Takumi Takeuchi, Tomomoto Ishikawa, Mika Handa, Tatsuya Miyake, Tsuyoshi Takiuchi, Tadashi Kimura

**Affiliations:** ^1^ Department of Reproductive Medicine Reproduction Clinic Tokyo Tokyo Japan; ^2^ Department of Obstetrics and Gynecology Graduate School of Medicine Osaka University Suita Japan; ^3^ Department of Reproductive Medicine Reproduction Clinic Osaka Osaka Japan; ^4^ Department of Clinical Genomics Graduate School of Medicine Osaka University Suita Japan

**Keywords:** advanced maternal age, endometrial receptivity test, personalized embryo transfer, recurrent implantation failure, window of implantation

## Abstract

**Purpose:**

To assess the clinical efficacy of personalized embryo transfer (pET) guided by a new endometrial receptivity test, ERPeak^SM^, in patients with recurrent implantation failure (RIF).

**Methods:**

Recurrent implantation failure patients of all ages at two private Japanese clinics from April 2019 to June 2020 were retrospectively analyzed. The intervention group (*n* = 244) received pET in accordance with endometrial receptivity testing results and was compared to control group (*n* = 306) receiving standardized timing, non‐personalized embryo transfer (npET). In propensity score matching analysis, the clinical pregnancy rate (CPR) and live birth rate (LBR) were compared between groups, and a subanalysis of advanced maternal age (AMA) (≥38 years old) versus non‐AMA (<38 years old) patients was also conducted.

**Results:**

The CPR and LBR of the pET group were significantly higher than those of the npET group (37.7% vs. 20.0%, adjusted OR: 2.64; 95%CI, 1.70–4.11, *p* < 0.001 and 29.9% vs. 9.7%, adjusted OR: 4.13; 95%CI, 2.40–7.13, *p* < 0.001, respectively). Furthermore, in the subanalyses, the CPR and LBR of the pET group were significantly higher than those of the npET group in both the AMA non‐AMA patients.

**Conclusions:**

The new ERPeak^SM^ endometrial receptivity test is a useful alternative diagnostic tool for poor‐prognosis patients, regardless of age.

## Graphical abstract

Personalized embryo transfer guided by a new endometrial receptivity test significantly improved clinical pregnancy rate and live birth rate in patients with recurrent implantation failure, regardless of age.

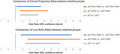

## 1 INTRODUCTION

Recurrent implantation failure (RIF) remains a challenging issue, although assisted reproductive technology (ART) has improved outcomes for struggling couples.^1^, ^2^ Aneuploid embryos are the major cause of implantation failure, especially in cases of advanced maternal age (AMA); however, several studies have demonstrated that even euploid blastocysts fail to implant in approximately 40% of transfers.^3^, ^4^, ^5^ The failure in implanting a euploid embryo suggests the synchronization between the embryo and the window of implantation (WOI) as another potential cause of RIF.^6^

Although transcriptomic diagnostic tools are popular, there remains limited evidence of the optimal indication of endometrial receptivity tests as well as conflicting effects reported on obstetric outcomes.^6^, ^7^, ^8^, ^9^, ^10^, ^11^, ^12^, ^13^, ^14^ A recent multicenter randomized controlled study investigated personalized embryo transfer (pET) guided by another widely used receptivity test found that the use of endometrial receptivity testing improved both cumulative live birth rates (per protocol) and cumulative pregnancy rates (intention‐to‐treat) after 1 year compared to standard frozen ET (FET) and fresh ET in good‐prognosis patients aged ≤37 years without RIF.^15^ Given the current situation in which the rate of AMA patients (aged ≥38 years) undergoing ART is up to 59.5% in Japan, and egg donation is not allowed, evaluating the effect of pET guided by an endometrial receptivity test on poor‐prognosis AMA patients with RIF is an essential and urgent issue.^16^, ^17^

While the receptivity testing conducted by Simón et al uses next‐generation sequencing to determine the transcriptomic profile of 248 genes, the new ERPeak^SM^ endometrial receptivity test analyzes 48 originally selected genes that are vastly distinct (only 7 in common) using real‐time quantitative polymerase chain reaction (RT‐qPCR), a method reported to have the highest sensitivity, widest dynamic range, and least bias for gene expression analysis.

This is the first report to investigate the efficacy of pET guided by the newly developed receptivity test. Furthermore, we aimed to demonstrate whether poor‐prognosis AMA patients with RIF can benefit from the endometrial receptivity test to determine its clinical indication.

## 2 MATERIALS AND METHODS

### 2.1 Patient characteristics

This retrospective cohort study examined obstetric outcomes from Japanese RIF patients (*N* = 1000) of all ages who received in vitro fertilization (IVF) care at two private Japanese fertility clinics between April 2019 and June 2020.

A RIF classification was determined in patients who failed to achieve clinical pregnancy with three or more IVF cycles in which one or two morphologically good‐quality blastocysts were transferred to the patient in each HRT or natural cycle.^1^, ^18^ Blastocysts of grade ≥3BB on Day 5 or 6 according to the Gardner scoring criteria were defined as good‐quality embryos.

All patients underwent the following infertility examinations: vaginal ultrasound, hysteroscopy, and endometrial biopsy for chronic endometritis. Pathology affecting the endometrial cavity, including hydrosalpinx, endometrial polyps, submucosal myomas, and chronic endometritis, was successfully treated prior to the period examined in this study. Patients were also examined for thyroid function and thrombophilia and were treated as required. All clinical data were retrieved from an electronic medical record system.

### 2.2 Endometrial preparation

All patients underwent HRT prior to endometrial biopsy (mock cycle, pET group only) or embryo transfer (transfer cycle, all patients). Estradiol (Premarin, transdermal patch, or both when necessary) was administered from Day 4 of menses. After identifying a trilaminar endometrium measuring ≥7.0 mm using transvaginal sonography within approximately 14 days following menses, we prescribed three types of progesterone (Utrogestan vaginal capsule 800 mg bid, Lutoral tablet 6 mg tid, and Progeston depot intramuscular injection 125 mg every 3 days) for all patients. We defined the first day of progesterone administration as “P + 0.”

### 2.3 Endometrial biopsy

Patients electing to pursue pET underwent a mock cycle of HRT as described above prior (1.8 ± 1.6 months; average ± SD) to their embryo transfer cycle. An endometrial biopsy was performed using a catheter called “ENDOSUCTION^®^” (Hakko Company, Ltd.) on Day P + 5 (113 h after progesterone impregnation) in the HRT cycle. We inserted the pipelle as far as the cavity. The endometrial sample was transferred to a cryotube containing RNAlater^®^ (Invitrogen) and shaken 10 times. It was then immediately stored at 4°C for at least 4 hours and shipped at ambient temperature (from 15 to 25°C) for endometrial receptivity testing.

### 2.4 Endometrial receptivity testing

Endometrial receptivity testing was developed and performed by a commercial genomics service laboratory (ERPeak^SM^, CooperSurgical, Inc). Initial development of the test has been described previously.^19^, ^20^ Briefly, 184 candidate genes were identified through targeted literature review of topics such as implantation, window of implantation, embryo attachment, proliferation, differentiation, and decidualization. The putative gene panel was then tested in a two‐arm study comparing the gene expression profiles of healthy donors (*N* = 96) and subfertile patients (*N* = 120) at LH + 2 and LH + 7. Of the 184 candidate genes, 85 showed significant differences in fold change between the study groups. Discriminant functional analysis paired with principal component analysis then demonstrated that 48 genes were found to explain >99.5% of total sample variance in estimating the receptivity status of the endometrium. After completion of panel selection, assay development (RNA isolation, reverse transcription, qRT‐PCR), and optimization of statistical classifiers, the assay was validated in an independent 173 endometrial biopsies, demonstrating robust accuracy (CooperSurgical, internal data).

### 2.5 Embryo transfer and retrospective analysis

Patients electing to undergo a standard npET underwent embryo transfer at P + 5 in an HRT cycle. Patients electing to undergo pET had their embryos transferred in accordance with the endometrial receptivity test results. Patients receiving a receptive classification received an embryo transfer that matched the timing of the biopsy in their previous mock HRT cycle (typically P + 5). Patients receiving a pre‐receptive or post‐receptive classification received embryo transfers 24 h after (typically P + 6) or in advance (typically P + 4), respectively, of their mock HRT cycle biopsy timing. No preimplantation genetic testing for embryo aneuploidy was performed, and no patients received oocyte donations due to Japanese ethical concerns. When multiple embryos were selected for transfer, they were transferred simultaneously in the same day (the planned ET date). We chose poor‐quality blastocyst as the second embryo in the pET and npET groups.

**FIGURE 1:**
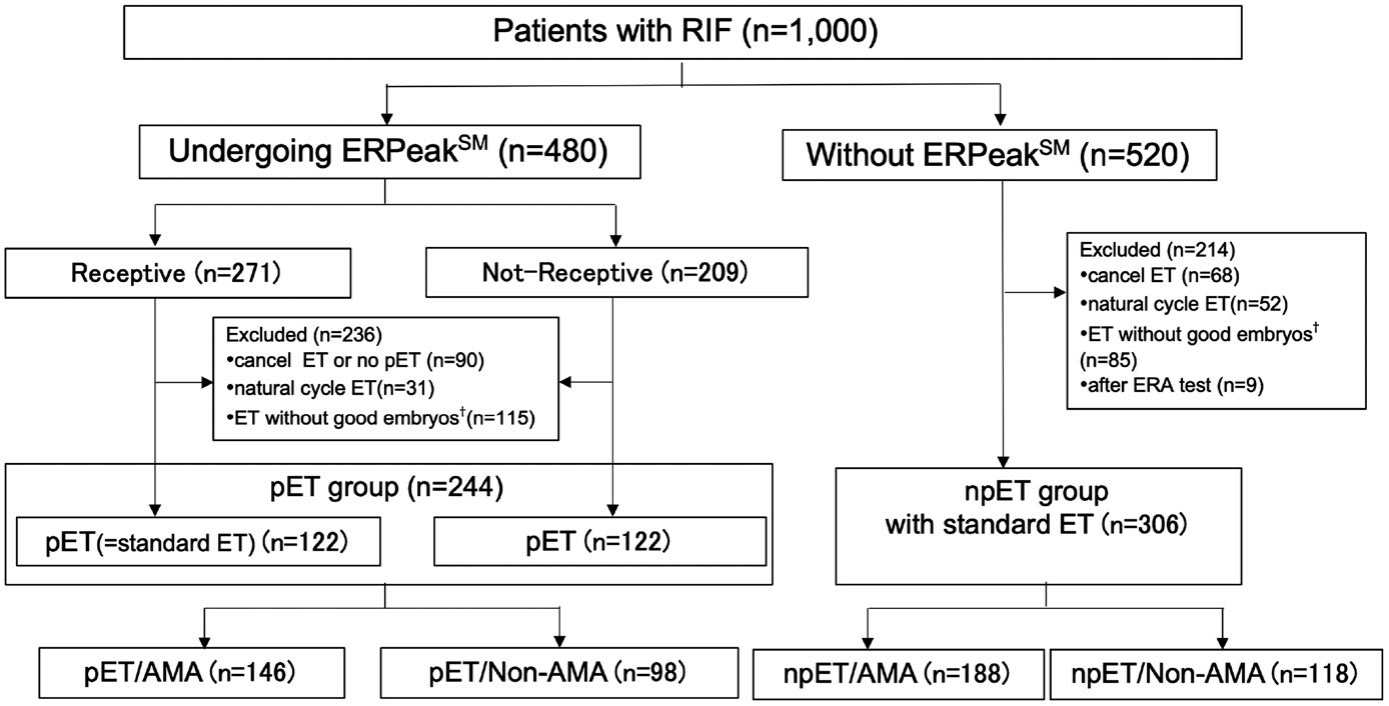
Distribution of included patients. AMA, advanced maternal age; ET, embryo transfer; npET, non‐personalized embryo transfer; pET, personalized embryo transfer; RIF, recurrent implantation failure. ^†^“Good embryo” means a blastocyst of grade ≥3BB according to the Gardner scoring criteria

Retrospective assessment of clinical outcomes was then performed. Certain exclusion criteria were assessed (Figure 1), including patients that: chose a natural hormone cycle rather than HRT, failed to produce good‐quality blastocysts, decided to forgo embryo transfer, elected standard npET timing rather than pET, or had previously received WOI testing only on an alternative platform.

Clinical outcomes were then compared between npET and pET groups, with subanalyses done comparing the role of AMA. Clinical pregnancy rates (CPR), miscarriage rates, and live birth rates (LBR) were documented. CPR were calculated as the total number of clinical pregnancies per ET cycles. Clinical pregnancies were defined by the presence of a gestational sac, including spontaneous abortions, while biochemical pregnancies and ectopic pregnancies were excluded. Miscarriage rates were calculated as the total number of spontaneous abortions before 13 weeks of gestation per the total number of clinical pregnancies. LBR were calculated as the total number of live births at >22 weeks' gestation per ETs.

### 2.6 Statistical analysis

The statistical analyses were performed using the chi‐square test or Fisher's exact test for comparisons of outcomes and the Mann–Whitney *U*‐test for comparisons of patient characteristics, with significance defined as *p* < 0.05.

Crude and adjusted odds ratios (ORs) and 95% confidence intervals (CIs) were calculated. Crude OR was determined by univariate logistic regression. Propensity score matching (PSM) was used to adjust for potential differences in characteristics between the pregnant and non‐pregnant patients using multiple logistic modeling. Propensity scoring was conducted using maternal age at ovum retrieval, anti‐Mullerian hormone (AMH) levels, maternal body mass index (BMI), gravidity, parity, infertility periods, the number of previous failed ET, and the number of transferred embryos in proportion of 1:1. EZR software version 4.1.1 (Saitama Medical Center, Jichi Medical University, Saitama, Japan) was used for statistical analysis.

## 3 RESULTS

### 3.1 Endometrial receptivity testing results

Of 1000 RIF patients, 480 (48.0%) underwent receptivity testing and 520 (52.0%) did not (Figure 1). The decision of whether to perform the receptivity test was made jointly by the patient and the physician. Within this pET group, 271 (55.4%) patients were found to be receptive, and 209 patients (44.6%) displayed a displaced window. Of the patients with a displaced window, 62.2% (130/209) indicated a pre‐receptive state and 37.8% (79/209) showed a post‐receptive state. A total of four patients (0.83%) required a second biopsy due to insufficient endometrial tissue, and the results of these biopsies were receptive in two patients and pre‐receptive in two patients. Eight patients wished to receive additional biopsies to confirm the precise WOI and repeated the receptivity test at 7, 9, 12, 15, 16, 19, 21, and 23 months later, respectively; 100% of the second biopsies showed the same receptivity status as their corresponding original biopsy.

### 3.2 Clinical outcomes of the pET and npET groups

Of 550 analyzed RIF patients who passed exclusion criteria (see Section 2) and underwent ET, 244 (average age: 38.2 ± 4.3 years, range: 20–45) were in the pET group and 306 (average age: 38.5 ± 4.1 years, range: 28–49) were in the npET group (Figure 1, Table 1). There were no significant differences in patient characteristics between the pET and npET groups except gravidity and parity. Of 244 cases in pET group, 22 with a history of pregnancy or childbirth with HRT (standard ET) were found to be a pre‐receptive or post‐receptive. Among 22 patients, eight patients (36.4%) got pregnant after undergoing pET.

**TABLE 1:** RIF patients’ profiles and reproductive outcomes before propensity score matching of the personalized embryo transfer (pET) and non‐personalized embryo transfer (npET) groups

	pET	npET	Crude odds ratio (95%CI)	*p*‐value
Patients: *N*	244	306	–	–
Age (years), mean ± SD	38.2 ± 4.3	38.5 ± 4.1	–	0.37
AMH (ng/ml), mean ± SD	3.00 ± 2.7	3.19 ± 3.1	–	0.44
BMI (kg/m^2^), mean ± SD	21.2 ± 2.4	20.9 ± 2.4	–	0.31
Gravida: *N* (%)	139 (57.0)	268 (87.6)	–	<0.001
Parity: *N* (%)	32 (13.1)	66 (21.6)	–	0.015
Infertility periods (months), mean ± SD	44.8 ± 22.6	42.3±23.0	–	0.21
No. of previous failed ET, mean ± SD	5.64 ± 2.7	5.83 ± 3.3	–	0.46
No. of transferred embryos per ET, mean ± SD	1.39 ± 0.5	1.41 ± 0.5	–	0.50
Clinical pregnancy rate: *N* (%)	92/244 (37.7)	59/306 (19.3)	2.53 (1.73−3.72)	<0.001
Miscarriage rate: *N* (%)	19/92 (20.7)	30/59 (50.8)	0.25 (0.12−0.52)	<0.001
Abnormal chromosomal rate of POC: *N* (%)	5/6 (83.3)	11/14 (78.6)	1.36 (0.11−16.6)	1.00
Live birth rate: *N* (%)	73/244 (29.9)	29/306 (9.5)	4.08 (2.55−6.53)	<0.001

Abbreviations: POC, products of conception; RIF, recurrent implantation failure; SD, standard deviation.

After the PSM analysis, reproductive outcomes (CPR and LBR) in the pET group were found to be significantly higher than those in the npET group (37.7% vs. 18.6%, adjusted OR: 2.64; 95% CI, 1.70–4.11, *p* < 0.001, and 29.8% vs. 9.3%, adjusted OR: 4.13; 95% CI, 2.40–7.13, *p* < 0.001, respectively). The miscarriage rate in the pET group was significantly lower than that in the npET group, whereas the chromosomal abnormality rates of the products of conception (POC) were similar between the two groups (Table 2).

**TABLE 2:** RIF patient outcomes in the personalized embryo transfer (pET) and non‐personalized embryo transfer (npET) groups after propensity score matching

	pET	npET	Adjusted odds ratio (95%CI)	*p*‐value
Propensity‐matched patients: *N*	215	215	–	–
Age (years), mean ± SD	38.5 ± 4.1	38.2 ± 4.3	–	0.45
AMH (ng/ml), mean ± SD	2.89 ± 2.7	3.18 ± 3.2	–	0.30
BMI (kg/m^2^), mean ± SD	21.1 ± 2.4	21.1 ± 2.4	–	0.92
Gravida: *N* (%)	132 (61.4)	131 (60.9)	–	0.87
Parity: *N* (%)	30 (14.0)	26 (12.1)	–	0.65
Infertility periods (months), mean ± SD	44.4 ± 23.3	44.2 ± 23.6	–	0.99
No. of previous failed ET, mean ± SD	5.64 ± 2.7	5.79 ± 3.4	–	0.55
No. of transferred embryos per ET, mean ± SD	1.41 ± 0.5	1.41 ± 0.5	–	0.87
Clinical pregnancy rate of: *N* (%)	81/215 (37.7)	40/215 (18.6)	2.64 (1.70−4.11)	<0.001
Miscarriage rate: *N* (%)	17/81 (21.0)	20/40 (50.0)	0.27 (0.12−0.60)	0.002
Abnormal chromosomal rate of POC: *N* (%)	4/6 (66.7)	8/10 (80.0)	0.50 (0.05−4.98)	0.60
Live birth rate: *N* (%)	64/215 (29.8)	20/215 (9.3)	4.13 (2.40−7.13)	<0.001

Abbreviations: POC, products of conception; RIF, recurrent implantation failure; SD, standard deviation.

Among the participants in the pET group, we also compared the clinical outcomes (CPR and LBR) after performing pET (standard ET) for receptive patients and pET (arrangement) for displaced WOI patients. There were no differences in the CPR and LBR between the two groups (40.5% vs. 45.2%; adjusted OR, 1.21; 95%CI, 0.66–2.24; *p* = 0.53; and 32.1% vs. 33.3%; adjusted OR, 1.06; 95%CI, 0.55–2.01; *p* = 0.87, respectively).

### 3.3 Clinical outcomes of the AMA and non‐AMA groups

Of 480 RIF patients who underwent receptivity testing, 326 were aged ≥38 years (average age: 41.8 ± 2.6 years, range: 38–49) and 154 were aged <38 years (average age: 34.0 ± 2.7 years, range: 20–37; Table 3). Although there was no significant difference in the frequency of displaced window detected by the receptivity test between the two groups, the rate of pre‐receptive status was higher in patients aged ≥38 years than in those aged <38 years (OR: 1.82; 95%CI, 1.00–3.31, *p* = 0.064).

**TABLE 3:** Receptivity statuses according to the receptivity testing of RIF patients aged ≥38 years and those aged <38 years

	Age ≥38 years	Age <38 years	Odds ratio (95%CI)	*p*‐value
Patients: *N*	326	154	–	–
No. of R/total analyzed	182/326 (55.8)	89/154 (57.8)	0.92 (0.63–1.36)	0.69
No. of NR/total analyzed	144/326 (44.2)	65/154 (42.2)	1.08 (0.74–1.60)	0.69
No. of pre‐receptive/NR	96/144 (66.7)	34/65 (52.3)	1.82 (1.00–3.31)	0.064
No. of post‐receptive/NR	48/144 (37.8)	31/65 (47.7)	0.55 (0.29–1.04)	0.064

Abbreviations: NR, not‐receptive; R, receptive; RIF, recurrent implantation failure.

**FIGURE 2:**
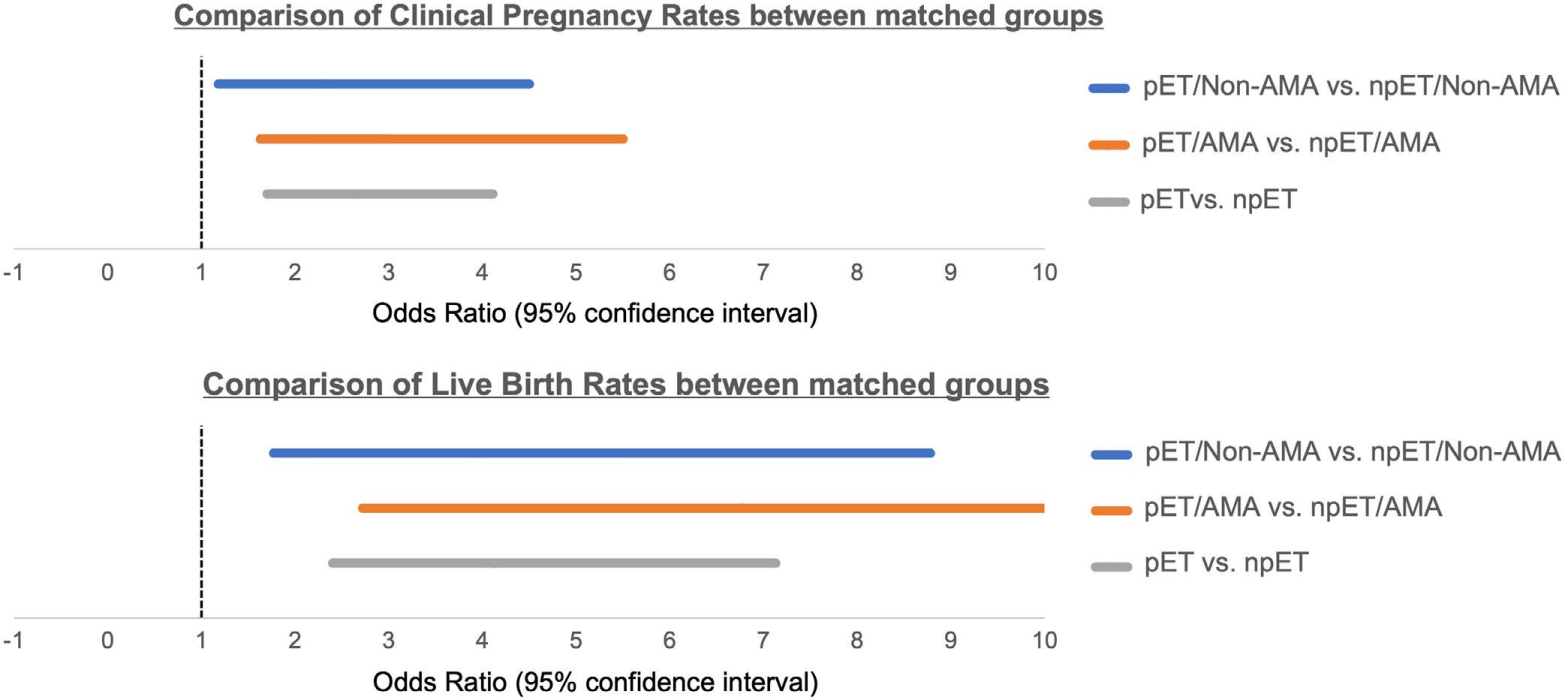
Odds ratio (95% confidence interval) for clinical pregnancy rates and live birth rates of matched subgroup patients. AMA, advanced maternal age; npET, non‐personalized embryo transfer; pET, personalized embryo transfer

Of 550 analyzed RIF patients who underwent pET or npET with morphologically good‐quality embryos, 334 (average age: 41.2 ± 2.1 years, range: 38–49) were in the AMA group and 216 (average age: 34.1 ± 2.6 years, range: 20−37) were in the non‐AMA group. After PSM, 82 pET/non‐AMA patients were matched to 82 npET/non‐AMA patients and 129 pET/AMA patients were matched to 129 npET/AMA patients. Under the PSM analysis, the CPR and LBR of the pET/non‐AMA group were significantly higher than those of the npET/non‐AMA group (42.7% vs. 24.4%, adjusted OR: 2.31; 95% CI, 1.18–4.50, *p* = 0.014, and 35.7% vs. 12.2%, adjusted OR: 3.94; 95% CI, 1.77–8.78, *p* < 0.001, respectively) (Table 4). Interestingly, the CPR and LBR of the pET/AMA group were also significantly higher than those of the npET/AMA group (34.1% vs. 14.7%, adjusted OR: 3.00; 95% CI, 1.63–5.50, *p* < 0.001, and 24.8% vs. 4.7%, adjusted OR: 6.76; 95% CI, 2.72–16.8, *p* < 0.001, respectively) (Table 5, Figure 2).

**TABLE 4:** RIF patient profiles and outcomes of the pET/non‐AMA and npET/non‐AMA groups after propensity score matching

	pET/non‐AMA	npET/non‐AMA	Adjusted odds ratio (95%CI)	*p*‐value
Propensity‐matched patients: *N*	82	82	–	–
Age (years), mean ± SD	34.4 ± 2.8	34.1 ± 2.4	–	0.44
AMH (ng/ml), mean ± SD	4.15 ± 3.0	4.10 ± 3.9	–	0.46
BMI (kg/m^2^), mean ± SD	20.9 ± 2.5	20.6 ± 2.2	–	0.57
Gravida: *N* (%)	38 (46.3)	34 (41.5)	–	0.75
Parity: *N* (%)	5 (6.1)	5 (6.1)	–	1.00
Infertility periods (months), mean ± SD	43.9 ± 21.4	42.9 ± 22.4	–	0.58
No. of previous failed ET, mean ± SD	5.41 ± 2.4	5.09 ± 3.1	–	0.053
No. of transferred embryos per ET, mean ± SD	1.37 ± 0.5	1.35 ± 0.5	–	0.87
Clinical pregnancy rate: *N* (%)	35/82 (42.7)	20/82 (24.4)	2.31 (1.18−4.50)	0.014
Miscarriage rate: *N* (%)	6/35 (17.1)	10/20 (50.0)	0.21 (0.05−0.84)	0.015
Abnormal chromosomal rate of POC: *N* (%)	1/2 (50.0)	3/6 (50.0)	–	1.00
Live birth rate: *N* (%)	29/82 (35.4)	10/82 (12.2)	3.94 (1.77−8.78)	<0.001

Abbreviations: AMA, advanced maternal age; npET, non‐personalized embryo transfer; pET, personalized embryo transfer; POC, products of conception; RIF, recurrent implantation failure.

**TABLE 5:** RIF patient profiles and outcomes of the pET/AMA and npET/AMA groups after propensity score matching

	pET/AMA	npET/AMA	Adjusted odds ratio (95%CI)	*p*‐value
Propensity‐matched patients: *N*	129	129	–	–
Age (years), mean ± SD	41.4 ± 1.9	41.0 ± 2.2	–	0.13
AMH (ng/ml), mean ± SD	2.18 ± 1.9	2.43 ± 2.2	–	0.43
BMI (kg/m^2^), mean ± SD	21.4 ± 2.2	21.4 ± 2.6	–	0.55
Gravida: *N* (%)	96 (74.4)	96 (74.4)	–	0.51
Parity: *N* (%)	24 (18.6)	28 (21.7)	–	0.53
Infertility periods (months), mean ± SD	45.1 ± 23.4	44.3 ± 21.1	–	0.86
No. of previous failed ET, mean ± SD	5.83 ± 2.8	5.93 ± 3.3	–	0.94
No. of transferred embryos per ET, mean ± SD	1.43 ± 0.5	1.38 ± 0.5	–	0.42
Clinical pregnancy rate: *N* (%)	44/129 (34.1)	19/129 (14.7)	3.00 (1.63−5.50)	<0.001
Miscarriage rate: *N* (%)	12/44 (27.3)	13/19 (68.4)	0.17 (0.05−0.56)	0.0043
Abnormal chromosomal rate of POC: *N* (%)	4/4 (100.0)	6/6 (100.0)	–	1.00
Live birth rate: *N* (%)	32/129 (24.8)	6/129 (4.7)	6.76 (2.72−16.8)	<0.001

Abbreviations: AMA, advanced maternal age; npET, non‐personalized embryo transfer; pET, personalized embryo transfer; POC, products of conception; RIF, recurrent implantation failure.

## 4 DISCUSSION

To our knowledge, this is the first report on the clinical outcomes of pET using the new ERPeak^SM^ endometrial receptivity test in RIF patients. pET guided by the receptivity test was effective for patients with RIF. Additionally, utilization of receptivity testing offered improved reproductive outcomes in both the AMA and non‐AMA groups.

This test analyzes 48 selected genes by RT‐qPCR. Due to its ability to measure minute amounts of nucleic acid within a sample in a linear fashion over many orders of magnitude of difference in concentrations,^21^ RT‐qPCR is widely considered the optimal method for gene expression quantification. One of the shortcomings of other widely utilized receptivity tests is a high retest rate. Previous studies report that 10.0%–36.4% of patients who received such testing results needed a second biopsy to detect the WOI.^11^, ^13^, ^22^, ^23^ In the current study, >99% of patients were given their results after just a single biopsy using the ERPeak^SM^ test. The robust nature and very high sensitivity of this platform is very important clinically, as it promotes taking smaller biopsies, minimizing any discomfort experienced by the patient. Additionally, very high success rates protect patients from needing to undergo an additional HRT cycle and biopsy, saving resources, time, and discomfort.

Interestingly, the selection and validation of the gene panel used in this endometrial receptivity test utilized samples from both fertile and subfertile women under both natural hormones and HRT, which contrasts with the development of other receptivity tests which utilize only fertile women during their natural menstrual cycle.^11^ Considering the differences in gene expression between fertile and infertile women as well as the difficulty of reproducibility in the natural menstrual cycle, the method of gene panel selection in ERPeak^SM^ may be reasonable.^13^, ^24^ Additionally, one concern in endometrial receptivity testing is whether the technology can consistently identify an accurate WOI using a biopsy obtained in a mock cycle when the transfer will be performed in a separate, subsequent cycle. To this point, it was very reassuring to see that 100% of patients (*N* = 8) who underwent follow‐up confirmatory testing with a second biopsy obtained in a different cycle (15.3 ± 5.7 months; average ± SD) had their original result confirmed, indicating a high degree of classification precision and physiological consistency. Moreover, 22 patients in the pET group had a history of pregnancy or childbirth with HRT (standard ET) that resulted in not‐receptive status detected by ERPeak^SM^ testing. The dynamic hormonal change during pregnancy or maternal aging might affect gene expression of the endometrium as it is a hormonally regulated organ.

In this study, pET guided by the ERPeak^SM^ endometrial receptivity test led to significantly improved obstetric outcomes in patients with RIF compared to outcomes in patients in the control group who did not undergo ERPeak^SM^ testing. Concerning the endometrial receptivity analysis (ERA) test, there have been limited studies reporting that pET guided by the test was superior to the standard ET without checking the receptivity statuses. Instead, several reports have shown similar clinical results after performing pET in displaced WOI patients and standard ET in receptive patients, concluding that the clinical outcomes of pET in those with non‐receptive endometrium increased to a level similar to that of the receptive patients.^22^, ^23^, ^25^ In 2020, Simon et al. published an RCT evaluating the reproductive outcomes of pET guided by the ERA tests. Interestingly, they showed a significant improvement in the cumulative live birth rate in patients undergoing pETs compared with that of frozen ETs (71.2% and 55.4%, *p* = 0.04). However, they did not show any differences in obstetric outcomes after the first ET.^15^ Besides, a single‐center cohort study reported that the live birth rates for the ERA and non‐ERA groups were not significantly different after performing propensity score matching.^14^ Another recent retrospective study concluded that the use of the ERA test in 488 women with RIF who underwent preimplantation genetic testing for aneuploidy (PGT‐A), ERA, or ERA+PGT‐A did not show any advantage.^26^ Although our study could not evaluate euploid embryo transfers, using this new endometrial receptivity test can have several benefits in patients with RIF. However, further RCTs of euploid pET based on ERPeak^SM^ testing are required.

To pursue the synchronization between an embryo and the WOI, the uniformity of patient characteristics and ET methods is of critical importance because several factors can influence endometrial gene expression, including chronic endometritis, BMI, and luteal support protocol.^27^, ^28^, ^29^ To eliminate these biases, all RIF patients in this study underwent endometrial biopsies for CD138 immunohistochemistry to detect chronic endometritis and, if diagnosed, were treated before receptivity testing. Additionally, no significant difference was found in BMI between the compared groups as shown in Table 1, although almost all patients were in the normal range (BMI 19–24.9 kg/m^2^). Furthermore, our standard programmed HRT cycle was conducted for all patients in both the mock cycle to obtain a biopsy and in the embryo transfer cycle, ensuring consistent endocrinology and gene network activation. We focused on FET with our unified programmed HRT with the same medications and excluded natural cycles in the current study to more precisely synchronize implantation timing of the blastocyst with the WOI, whereas many studies utilizing other receptivity tests included ET with natural cycles. In natural cycles, it is more difficult to control the precise time when progesterone starts to rise compared to HRT cycles. A premature rise in progesterone and shifted endometrial secretory transformation can occur in the natural cycle and result in a shifted WOI, leading to dyssynchrony between an embryo and the endometrium.^24^ Electing to forgo these added measures might be one of the reasons why the efficacy of other receptivity tests is still controversial in recent studies.^9^, ^14^

We also compared the data of patients in the pET (standard ET) and pET (arrangement) groups and found that there were no differences in clinical outcomes (CPR and LBR), in line with the findings of previous studies that used the ERA test.^22^, ^23^ That the receptive patients can also benefit from ERPeak^SM^ testing might be attributed to endometrial scratching which is a technique proposed to facilitate embryo implantation. A previous large RCT showed the same LBRs in the scratch and control groups and failed to find a positive effect in a subgroup analysis of women with ≥2 IVF failures.^30^ However, another RCT reported that women with three or more previous implantation failures presented a significant increase in clinical pregnancy rate after scratching.^31^ As we defined RIF as having three or more implantation failures, scratching by endometrial biopsy might have affected the clinical outcome in this study.

In the current study, the miscarriage rate in RIF patients was significantly reduced by pET when compared to npET. A previous retrospective cohort study reported that pregnancies by ET with Day 2 donated embryos were achieved following 2–5 days of progesterone administration, with the optimal WOI being after 3–4 days of progesterone.^32^ The idea of an optimal WOI is supported by a subsequent study that demonstrated that delayed implantation on the edge of the WOI can lead to an increased risk of early pregnancy loss due to abnormal placentation.^33^, ^34^ In our study, the rates of abnormal chromosomal detection in the POC were similar between the pET and npET groups. If the endometrial receptivity test could reduce the miscarriage rate rather than chromosomal abnormality, the chromosomal abnormality rate would have been higher in the pET group than in the npET group. However, we could not find any differences on the chromosomal abnormality rate between the two groups. This could be attributed to the limited number of the cases receiving POC. A recent randomized control study also suggested that inadequate progesterone supplementation exposure time can cause early pregnancy loss because of the insufficient decidualization of the endometrium.^35^ Thus, performing pET at the optimal WOI may be important to prevent early pregnancy loss and improve obstetric outcomes.

To the best of our knowledge, there have been no other reports on the clinical characteristics and outcomes of pET focused on AMA patients. According to the ERPeak^SM^ endometrial receptivity test, approximately 40% of RIF patients had a displaced WOI in both patients aged ≥38 years and those <38 years, which is relatively consistent with the findings of previous studies that reported not‐receptive rates of 40.0% and 45.7%, respectively, after using other receptivity tests.^7^, ^14^ Interestingly, in the pET patients found to have a displaced window, a higher percentage of pre‐receptive state was observed in patients aged ≥38 years than in those aged <38 years, suggesting that the WOI could be displaced backwards in older patients (Table 3). On the contrary, post‐receptive patients were more frequently observed in ERPeak^SM^ test compared to the ERA test. The misalignment of receptivity status between the two tests might be caused by differences of used platforms and gene panels.

Notably, obstetric outcomes in both the AMA group and the non‐AMA group were significantly improved by pET guided by these receptivity results. This would indicate that pET is beneficial, even for AMA patients with RIF. Interestingly, the pET/AMA group had better clinical results (i.e., CPR and LBR) compared to the npET/Non‐AMA group, suggesting that chromosomal abnormalities due to an age factor and an endometrial factor might have been major causes of IVF failures. Besides, we assumed that non‐AMA patients with RIF would have other unsolvable factors, such as an immune factor, other than the embryo or endometrial factor, resulting in poor outcomes.^2^ These might be the reasons why the pET/AMA group presented better reproductive outcomes compared to the npET/Non‐AMA group. While a randomized control trial by Simon. et al in 2020 was well designed, they only selected good‐prognosis patients aged ≤37 years without RIF.^15^ Although chromosomal abnormality is thought to be the major cause of implantation failure for AMA patients, we should reconsider the synchronization between the embryo and the WOI in AMA patients as possible major cause of RIF. A further randomized control study of euploid pET in AMA patients with RIF is required.

This is the first cohort using a PSM analysis to compare RIF patients undergoing the new endometrial receptivity test, ERPeak^SM^, after failed ETs with RIF patients whose endometrial receptivity was not assessed. Other strengths of this study include the large sample size and the unified luteal support protocol in an HRT cycle, which can lead to stable endometrial gene expression.^13^, ^29^ There are several limitations, including the retrospective nature of the study. Some hypothesize that minor endometrial trauma (e.g., endometrial scratching) may impact embryo implantation, and this phenomena was not tested here with a patient group that underwent biopsy but did not receive testing. Embryos were selected for transfer by morphology alone, rather than by chromosomal screening, which may have affected clinical outcomes. Further investigations using euploid embryos are needed to confirm the efficacy of this improved endometrial receptivity test. pET guided by an endometrial receptivity test can be a useful protocol to compensate for the failed implantation of euploid embryos, even in AMA patients.

Our data support the clinical use of a new endometrial receptivity test in patients with RIF. For the first time, we show clinical evidence that pET guided by the ERPeak test can be useful in the treatment of RIF patients, including those with AMA, whereas further consideration will be needed to determine whether the same result is obtained in pET using euploid embryos.

## CONFLICTS OF INTEREST

The authors declare that they have no conflict of interest.

## ETHICAL APPROVAL STATEMENT

This study was approved by Reproduction Clinic Tokyo Review Board on April 2, 2021 (Approval no: 2021–1). *Clinical trial Registry*: Not applicable. This study was a retrospective observational study.

## HUMAN RIGHTS STATEMENTS AND INFORMED CONSENT

All procedures followed were in accordance with the ethical standards of the responsible committee on human experimentation (institutional and national) and with the Helsinki Declaration of 1964 and its later amendments. Written informed consent was obtained from all patients before endometrial testing. The data that support the findings of this study are available from the corresponding author upon reasonable request.

## ANIMAL STUDIES

This article does not contain any study with animal participants.

## References

[1] Busnelli A , Somigliana E , Cirillo F , Baggiani A , Levi‐Setti PE . Efficacy of therapies and interventions for repeated embryo implantation failure: a systematic review and meta‐analysis. Sci Rep. 2021;11:1747. doi:10.1038/s41598-021-81439-6 33462292 PMC7814130

[2] Bashiri A , Halper KI , Orvieto R . Recurrent implantation failure‐update overview on etiology, diagnosis, treatment and future directions. Reprod Biol Endocrinol. 2018;16:121. doi:10.1186/s12958-018-0414-2 30518389 PMC6282265

[3] Franasiak JM , Forman EJ , Hong KH , et al. The nature of aneuploidy with increasing age of the female partner: a review of 15,169 consecutive trophectoderm biopsies evaluated with comprehensive chromosomal screening. Fertil Steril. 2014;101:656‐63.e1. doi:10.1016/j.fertnstert.2013.11.004 24355045

[4] Mikwar M , MacFarlane AJ , Marchetti F . Mechanisms of oocyte aneuploidy associated with advanced maternal age. Mutat Res. 2020;785:108320. doi:10.1016/j.mrrev.2020.108320 32800274

[5] Cimadomo D , Capalbo A , Dovere L , et al. Leave the past behind: women's reproductive history shows no association with blastocysts’ euploidy and limited association with live birth rates after euploid embryo transfers. Hum Reprod. 2021;36:929‐940. doi:10.1093/humrep/deab014 33608730

[6] Teh WT , McBain J , Rogers P . What is the contribution of embryo‐endometrial asynchrony to implantation failure? J Assist Reprod Genet. 2016;33:1419‐1430. doi:10.1007/s10815-016-0773-6 27480540 PMC5125144

[7] Eisman LE , Pisarska MD , Wertheimer S , et al. Clinical utility of the endometrial receptivity analysis in women with prior failed transfers. J Assist Reprod Genet. 2020;38:645‐650. doi:10.1007/s10815-020-02041-9 PMC7910393 33454901

[8] Riestenberg C , Kroener L , Quinn M , Ching K , Ambartsumyan G . Routine endometrial receptivity array in first embryo transfer cycles does not improve live birth rate. Fertil Steril. 2021;115:1001‐1006. doi:10.1016/j.fertnstert.2020.09.140 33461752

[9] Bassil R , Casper R , Samara N , et al. Does the endometrial receptivity array really provide personalized embryo transfer? J Assist Reprod Genet. 2018;35:1301‐1305. doi:10.1007/s10815-018-1190-9 29737471 PMC6063827

[10] Díaz‐Gimeno P , Ruiz‐Alonso M , Blesa D , et al. The accuracy and reproducibility of the endometrial receptivity array is superior to histology as a diagnostic method for endometrial receptivity. Fertil Steril. 2013;99:508‐517. doi:10.1016/j.fertnstert.2012.09.046 23102856

[11] Tan J , Kan A , Hitkari J , et al. The role of the endometrial receptivity array (ERA) in patients who have failed euploid embryo transfer. J Assist Reprod Genet. 2018;35:683‐692. doi:10.1007/s10815-017-1112-2 29327111 PMC5949105

[12] Nalini M . Endometrial receptivity array: clinical application. J Hum Reprod Sci. 2015;8:121‐129. doi:10.4103/0974-1208.165153 26538853 PMC4601169

[13] Ben Rafael Z . Endometrial Receptivity Analysis (ERA) test: an unproven technology. Hum Reprod Open. 2021;2021:hoab010. doi:10.1093/hropen/hoab010 33880419 PMC8045470

[14] Bergin K , Eliner Y , Duvall DW Jr , et al. The use of propensity score matching to assess the benefit of the endometrial receptivity analysis in frozen embryo transfers. Fertil Steril. 2021;116:396‐403. 33926718 10.1016/j.fertnstert.2021.03.031

[15] Simón C , Gómez C , Cabanillas S , et al. A 5‐year multicentre randomized controlled trial comparing personalized, frozen and fresh blastocyst transfer in IVF. Reprod Biomed Online. 2020;41:402‐415. doi:10.1016/j.rbmo.2020.06.002 32723696

[16] Japan Society of Obstetrics and Gynecology . ART Registry of Japan, 2008. Accessed September 23, 2021. https://plaza.umin.ac.jp/~jsog‐art/ART%20Registry%20of%20Japan,2008.pdf

[17] Nakagawa K , Kuroda K , Sugiyama R . Clinical strategies for ART treatment of infertile women with advanced maternal age. Reprod Med Biol. 2019;18(1):27‐33. doi:10.1002/rmb2.12240 30814909 PMC6378758

[18] Simon A , Laufer N . Repeated implantation failure: clinical approach. Fertil Steril. 2012;97:1039‐1043. doi:10.1016/j.fertnstert.2012.03.010 22464086

[19] ERPeakSM endometrial receptivity test. Accessed May 31, 2021. https://fertility.coopersurgical.com/genomics/erpeak‐endometrial‐receptivity‐test/

[20] Enciso M , Carrascosa JP , Sarasa J , et al. Development of a new comprehensive and reliable endometrial receptivity map (ER Map/ER Grade) based on RT‐qPCR gene expression analysis. Hum Reprod. 2018;33:220‐228. doi:10.1093/humrep/dex370 29315421

[21] Bustin SA . Absolute quantification of mRNA using real‐time reverse transcription polymerase chain reaction assays. J Mol Endocrinol. 2000;25(2):169‐193. 11013345 10.1677/jme.0.0250169

[22] Hashimoto T , Koizumi M , Doshida M , et al. Efficacy of the endometrial receptivity array for repeated implantation failure in Japan: a retrospective, two‐centers study. Reprod Med Biol. 2017;16:290‐296. doi:10.1002/rmb2.12041 29259480 PMC5715887

[23] Ruiz‐Alonso M , Blesa D , Díaz‐Gimeno P , et al. The endometrial receptivity array for diagnosis and personalized embryo transfer as a treatment for patients with repeated implantation failure. Fertil Steril. 2013;100:818‐824. doi:10.1016/j.fertnstert.2013.05.004 23756099

[24] Lessey BA , Young SL . What exactly is endometrial receptivity? Fertil Steril. 2019;111:611‐617. doi:10.1016/j.fertnstert.2019.02.009 30929718

[25] Patel JA , Patel AJ , Banker JM , Shah SI , Banker MR . Personalized embryo transfer helps in improving in vitro fertilization/ICSI outcomes in patients with recurrent implantation failure. J Hum Reprod Sci. 2019;12(1):59‐66. 31007469 10.4103/jhrs.JHRS_74_18 PMC6472200

[26] Cozzolino M , Diaz‐Gimeno P , Pellicer A , Garrido N . Evaluation of the endometrial receptivity assay and the preimplantation genetic test for aneuploidy in overcoming recurrent implantation failure. Assist Reprod Genet. 2020;37:2989‐2997. 10.1007/s10815-020-01948-7 PMC7714804 32974805

[27] Comstock IA , Diaz‐Gimeno P , Cabanillas S , et al. Does an increased body mass index affect endometrial gene expression patterns in fertile patients? A functional genomics analysis. Fertil Steril. 2017;107:740‐8.e2. doi:10.1016/j.fertnstert.2016.11.009 27919438

[28] Kuroda K , Horikawa T , Moriyama A , et al. Impact of chronic endometritis on endometrial receptivity analysis results and pregnancy outcomes. Immun Inflamm Dis. 2020;8:650‐658. doi:10.1002/iid3.354 32969185 PMC7654412

[29] Bermejo A , Cerrillo M , Ruiz‐Alonso M , et al. Impact of final oocyte maturation using gonadotropin‐releasing hormone agonist triggering and different luteal support protocols on endometrial gene expression. Fertil Steril. 2014;101:138‐46.e3. doi:10.1016/j.fertnstert.2013.09.033 24182413

[30] Lensen S , Osavlyuk D , Armstrong S , et al. A randomized trial of endometrial scratching before in vitro fertilization. N Engl J Med. 2019;380:325‐334. 30673547 10.1056/NEJMoa1808737

[31] Olesen MS , Benedicte Hauge B , Ohrt L , et al. Therapeutic endometrial scratching and implantation after IVF: a multicenter randomized controlled trial. Fertil Steril. 2019;112:1015‐1021. 31843072 10.1016/j.fertnstert.2019.08.010

[32] Prapas Y , Prapas N , Jones EE , et al. The window for embryo transfer in oocyte donation cycles depends on the duration of progesterone therapy. Hum Reprod. 1998;13:720‐723. doi:10.1093/humrep/13.3.720 9572441

[33] Wilcox AJ , Baird DD , Weinberg CR . Time of implantation of the conceptus and loss of pregnancy. N Eng J Med. 1999;340:1796‐1799. 10.1056/NEJM199906103402304 10362823

[34] Franasiak JM , Scott RT Jr . Recurrent Implantation Failure: Etiologies and Clinical Management. Springer; 2018.

[35] van de Vijver A , Polyzos NP , Van Landuyt L , et al. What is the optimal duration of progesterone administration before transferring a vitrified‐warmed cleavage stage embryo? A randomized controlled trial. Hum Reprod. 2016;31:1097‐1104. 27005893 10.1093/humrep/dew045

